# The journey of personalizing gastric cancer treatment

**DOI:** 10.1186/s40880-016-0149-4

**Published:** 2016-08-31

**Authors:** Li Yan

**Affiliations:** 1 US Chinese Anti-Cancer Association, Martinez, CA 94553 USA; 2 Beijing Cancer Hospital and Institute, Peking University School of Oncology, Beijing, 100142 P. R. China

**Keywords:** Gastric cancer, Surgery, Molecular characterization, Personalized medicine

## Abstract

Gastric cancer ranks the fourth most prevalent malignancy yet it is the second leading cause of cancer-related death. Every year, gastric cancer adds nearly 1 million new cancer cases, and 723,000 or 10% of cancer deaths to the global cancer burden. Approximately, 405,000 or 43% of the new cases and 325,000 or 45% of the deaths are in China, making gastric cancer a particularly challenging malignancy. This thematic series discusses the molecular classifications of gastric cancer by the Cancer Genome Atlas (TCGA) and the Asian Cancer Research Group (ACRG) as well as the implications in personalized therapeutic choices; discusses the evolution of gastric surgery and presents perspectives on surgical techniques in treating gastric cancer; and reviews current and emerging targeted agents as well as immunotherapies in treating gastric cancer. With these advancements in molecular characterization, surgical intervention, and targeted and immunotherapies, gastric cancer will enter a personalized medicine era in the next 5 years.

Gastric (including gastroesophageal junction, GEJ) cancer ranks the fourth most prevalent malignancy yet it is the second leading cause of cancer-related death worldwide. Every year, gastric cancer adds nearly 1 million new cancer cases, and accounts for 10% of cancer deaths to the global cancer burden [1]. Approximately, 405,000 or 43% of the new cases and 325,000 or 45% of gastric cancer-related deaths are in China, making gastric cancer a particularly challenging malignancy [1]. On the contrary, the overall incidence of gastric cancer in the western world has been steadily declining, except for GEJ cancer [2]. Because of the difference in disease prevalence between the developed and developing countries, gastric cancer has been a dormant indication in terms of basic research, translational medicine, as well as new drug development.

Surgery has remained as the only potential curative treatment option for early-stage gastric cancer. Over the past two decades, the surgical management of gastric cancer has evolved substantially. Shiozaki et al. [3] summarized the key improvements in gastric cancer surgery in this period of time. Four key areas were investigated in carefully designed randomized controlled studies to include comparisons between D1 or D2 lymph node dissection in total or subtotal gastrectomy; gastrectomy with or without splenectomy or pancreatectomy along with D2 dissection; total or subtotal gastrectomy with or without bursectomy; and the use of laparoscopic surgery in treating gastric cancer. Some of these trials assessed the impact of D1 versus D2 dissection on long-term survival. These studies provided critical evidence for surgeons to consider while selecting different surgical options based on individual patient conditions.

Unfortunately, the vast majority of patients with gastric cancer are either diagnosed with advanced unresectable or metastatic diseases or suffered with disease recurrence after initial surgical resection. In the era of targeted therapy, treatment of advanced-stage gastric cancer has been lagging behind. Chemotherapy remains as the only option for the majority of patients with moderate survival benefits. Two main intertwining causes underlie this unsatisfying situation: the lack of research and development incentives by western pharmaceutical companies in this disease and the gaps in understanding of this complex disease at the molecular biology level. As a disease that plaques population in developing world, gastric cancer did not receive the same attention as other cancer types such as lung, breast, colorectal, and prostate cancers did. In the past two decades, only a few targeted drugs were tested in this disease. On the other hand, the slow pace in discovering novel therapeutics is in part due to the complexity of gastric cancer.

In recent years, we have witnessed substantial advancement in research and development efforts in gastric cancer. The molecular characterization of gastric cancer paved the path for increased investment in finding effective treatments for this disease. Two articles in this series review the seminal work by the Asian Cancer Research Group (ACRG) [4] and by the Cancer Genome Atlas (TCGA) [5]. In contrary to the traditional pathological classification of gastric cancer into diffuse and intestinal subtypes, ACRG and TCGA use gene expression profile to classify gastric cancer into different subtypes based on common genomic alterations. These efforts began to provide a more comprehensive understanding of the high heterogeneity and diverse pathobiology of gastric cancer. These molecular data together with longitudinal clinical data now divide gastric cancer into more distinctive subtypes. Each subtype carries a set of defined genomic alterations, and some of them are targetable. Such knowledge enables more effective drug development based on personalized medicine approaches.

In the review article by Sun and Yan [6], recent advances in discovering and developing innovative cancer therapies were analyzed in the context of their potential influence in clinical management of advanced-stage gastric cancer. With the introduction of the first targeted therapy, e.g., trastuzumab [an anti-HER2 (v-erbB2 avian erythroblastic leukemia viral oncogene homolog 2) antibody], gastric cancer entered the era of personalized medicine following many other tumor types. Although only patients with strong HER2 overexpression or gene amplification benefit from this targeted treatment, it represents the first transformational change in gastric cancer treatment in two decades. Following trastuzumab, ramucirumab, a monoclonal antibody vascular endothelia growth factor receptor-2 (VEGFR-2) antagonist, demonstrated survival benefits in both first- and second-line treatment of gastric cancer. In parallel, apatinib, an oral small-molecule VEGFR-2 tyrosine kinase inhibitor (TKI), also significantly prolonged survival in patients with advanced gastric cancer whose disease progressed after first- or second-line chemotherapy treatment. More recently, anti-programmed cell death protein 1 (PD-1)/programmed death-ligand 1 (PD-L1) immunotherapies are generating encouraging preliminary results either as monotherapy or in combination with chemotherapies in gastric cancer. It is anticipated that in the next few years, significant changes will be seen in the landscape of gastric cancer clinical treatment when multiple late-phase clinical trials with these immunotherapies read out. Gastric cancer will enter into a new era of personalized medicine.
